# Chemical Imaging of Hierarchical Porosity Formation within a Zeolite Crystal Visualized by Small‐Angle X‐Ray Scattering and In‐Situ Fluorescence Microscopy

**DOI:** 10.1002/anie.202101747

**Published:** 2021-05-05

**Authors:** Matthias Filez, Martin Vesely, Ivan Garcia‐Torregrosa, Marianna Gambino, Özgün Attila, Florian Meirer, Eugene A. Katrukha, Maarten B. J. Roeffaers, Jan Garrevoet, Lukas C. Kapitein, Bert M. Weckhuysen

**Affiliations:** ^1^ Inorganic Chemistry and Catalysis Debye Institute of Nanomaterials Science Utrecht University Universiteitsweg 99 3584CG Utrecht The Netherlands; ^2^ Cell Biology, Neurobiology and Biophysics Faculty of Science Utrecht University Padualaan 8, 3584 CH Utrecht The Netherlands; ^3^ Centre for Membrane Separations, Adsorption, Catalysis and Spectroscopy for Sustainable Solutions (cMACS) Department of Microbial and Molecular Systems KU Leuven Celestijnenlaan 200F 3001 Leuven Belgium; ^4^ Deutsches Elektronen-Synchrotron DESY Notkestrasse 85 22607 Hamburg Germany

**Keywords:** desilication, hierarchical nanoporosity, in situ fluorescence microscopy, small-angle X-ray scattering microscopy, zeolite

## Abstract

Introducing hierarchical porosity to zeolites is vital for providing molecular access to microporous domains. Yet, the dynamics of meso‐ and macropore formation has remained elusive and pore space ill‐characterized by a lack of (in situ) microscopic tools sensitive to nanoporosity. Here, we probe hierarchical porosity formation within a zeolite ZSM‐5 crystal in real‐time by in situ fluorescence microscopy during desilication. In addition, we introduce small‐angle X‐ray scattering microscopy as novel characterization tool to map intracrystal meso‐ and macropore properties. It is shown that hierarchical porosity formation initiates at the crystal surface and propagates to the crystal core via a pore front with decreasing rate. Also, hierarchical porosity only establishes in specific (segments of) subunits which constitute ZSM‐5. Such space‐dependent meso‐ and macroporosity implies local discrepancies in diffusion, performance and deactivation behaviors even within a zeolite crystal.

## Graphical abstract

Hierarchical porosity within a zeolite crystal is visualized by X‐ray scattering microscopy to probe nanoporosity within the inorganic zeolite phase. In complement, in situ fluorescence microscopy allows to monitor pore hierarchization in real‐time via organic fluorescent stains, which fill the meso‐ and macropore space.

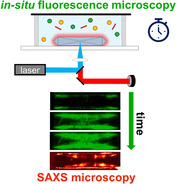

Zeolite catalysts are microporous crystalline aluminosilicates, displaying unique shape selectivity through their steric pore space.[^1^, ^2^] However, such selectivity requires molecularly‐sized pores that impose severe diffusion limitations on molecular transport towards Brønsted acid sites.[^3^, ^4^] Diverse strategies have been developed to introduce auxiliary meso‐ (2–50 nm) and macroporosity (>50 nm) for enhancing molecular transport towards microporous domains. These methods include bottom‐up approaches, for example, by using templates or nanocrystal synthesis, or more conventional and industrially applied top‐down routes, such as steaming, acid leaching and desilication, all yielding so‐called “hierarchical porosity”.[^5^, ^6^]

Lately, an advanced set of characterization tools has been developed to study the pore space architecture of hierarchical zeolites.[^7^, ^8^, ^9^, ^10^] However, none of the explored tools can spatially map meso‐/macroporosity across μm‐crystals without sample destruction and monitor pore formation in situ. For example, N_2_/Ar‐adsorption, Hg‐porosimetry^[11]^ and positron annihilation lifetime spectroscopy^[12]^ have proven valuable for determining pore size distributions and interconnectivity, but are bulk techniques. (Cryo)transmission electron microscopy[^12^, ^13^] and tomography[^14^, ^15^, ^16^] provide atomic resolution, but typically image nm‐scale volumes, while focused‐ion beam scanning electron microscopy^[17]^ scans large volumes but is sample destructive and non‐sensitive to small mesopores. X‐ray nanotomography[^18^, ^19^] offers non‐destructive sampling of μm‐scale volumes, but has 20 nm spatial resolution not detailing mesoporosity. The design of next‐generation hierarchical zeolites thus relies on the advent of (in situ) microscopic tools sensitive to meso‐ and macroporosity, which can scan across large crystal volumes.

Here, we present the first application of in situ fluorescence microscopy to follow‐up on the dynamics of hierarchical porosity formation within a zeolite ZSM‐5 crystal during desilication. Fluorescence microscopy probes organic dyes, which stain the meso‐ and macropore walls and allows fast charting of hierarchical porosity in the zeolite 3D volume.[^20^, ^21^] In complement, we introduce currently‐unexplored small‐angle X‐ray scattering (SAXS) microscopy as a direct characterization tool to chart and speciate the meso‐ and macropore properties of the inorganic zeolite phase with nm‐scale sensitivity.

The ZSM‐5 crystals here studied have large coffin‐shape and constitute six subunits: 2 longitudinal and 4 pyramidal (Figure 1 a), intergrown at their interface.[^21^, ^22^] Amongst the pyramidal, (100)′‐oriented “gable” subunits are crystallographically 90 ° rotated compared to the (100) “roof” subunits around their common c‐axis. All pyramidal subunits thus expose sinusoidal pores at the crystal surface, while straight pores run parallel below the surface.

**Figure 1:**
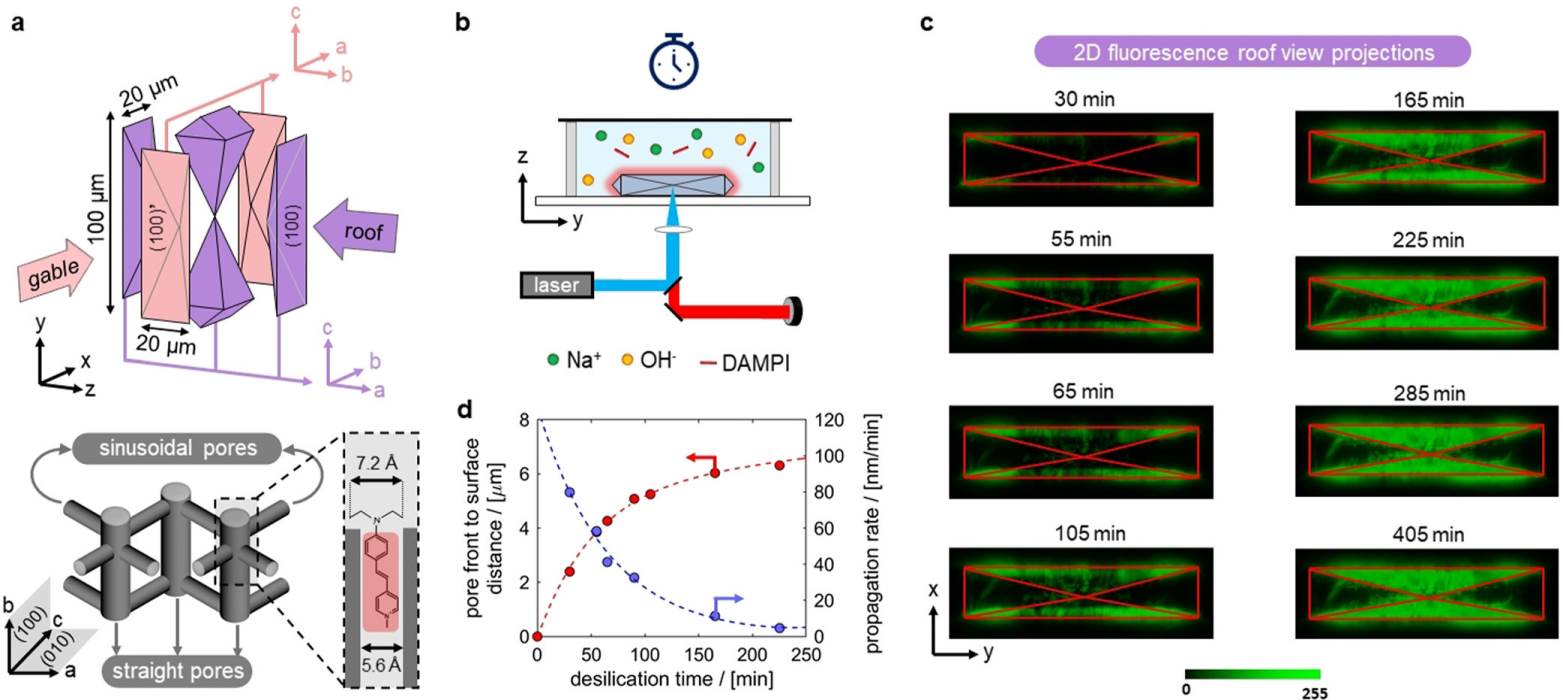
a) Subunit structure and micropore orientation of ZSM‐5. DAMPI fluorescent dye narrowly fits in straight channel (5.6 Å) of ZSM‐5, while its terminal diethylamino‐group (7.2 Å) blocks further intrusion into the micropore. b) in situ fluorescence microscopy to monitor desilication by mixing ZSM‐5 in a reaction cell with 0.2 M NaOH solution containing DAMPI. The laser raster scans a ZSM‐5 crystal (roof view). c) 2D in situ fluorescence projections (roof view) averaged over the *z*‐heights of the crystal during hierarchical porosity formation. d) pore front position relative to the external surface of gable pyramidal subunits (left axis) and propagation rate of pore formation (right axis) vs. desilication time.

To monitor the dynamics of desilication and pore hierarchy formation, first, a cell containing ZSM‐5 crystals was filled with a 0.2 M NaOH solution, containing 2 μM DAMPI fluorescent dyes (4‐(4‐diethylaminostyryl)‐N‐methylpyridinium‐iodide, SI‐S1). These dyes narrowly fit in straight channels of ZSM‐5, while its terminal diethylamino‐group (7.2 Å) blocks further intrusion into straight micropores. DAMPI therefore selectively visualizes zones of the crystal surface giving access to straight channels.^[21]^ After sealing, in situ confocal fluorescence 3D maps were collected during desilication (40 °C, 6.3 hours) by xy‐raster scanning a ZSM‐5 crystal in roof view at different *z*‐heights (Figure 1 b, SI‐S2, 488 nm, circular polarization). 2D fluorescence projections in roof view were obtained by summing xy‐maps over all *z*‐heights of the crystal, yielding a time‐resolved evolution of meso‐/macropore formation throughout the crystal volume (Figure 1 c). A progressive development of hierarchical porosity is manifested over time, initiating at the crystal external surface and gradually propagating via a pore front to the crystal core with decreasing propagation rate (Figure 1 d, SI‐S3). These observations do not occur when a 2 μM DAMPI solution is added without 0.2 M NaOH (SI‐S4) and cannot originate from diffusion limitations of DAMPI probes into the zeolite, since DAMPI diffusion into zeolites with pre‐formed hierarchical porosity established within ≈120 seconds (SI‐S5). Remarkably, meso‐/macropore generation is only observed in selected regions of the crystal, that is, in projected areas of gable subunits of zeolite ZSM‐5.

To unravel this location dependency of porosity formation, inspection of the fluorescence maps inside the crystal volume is required. Therefore, the xy‐maps at an intermediate phase of desilication are shown for different *z*‐heights throughout the ZSM‐5 half crystal (Figure 2 b, *z*=0–10 μm). At the crystal's external surface (*z*=0 μm), the confocal plane images the base of the roof pyramid only. With increasing *z*‐values, cross‐sections cut through 5 subunits and evolve to the crystal's mid‐plane (*z*=10 μm) where sections of gable pyramidal and longitudinal subunits are exposed only (Figure 2 a). Hierarchical porosity clearly forms throughout the entire gable pyramidal subunits, while the longitudinal subunits are robust against pore formation and remain meso‐/macropore‐free (SI‐S6). Surprisingly, only selected triangular regions within the roof pyramidal subunits develop hierarchical porosity (Figure 2 b,c). 3D reconstructions are shown in SI‐S6.

**Figure 2:**
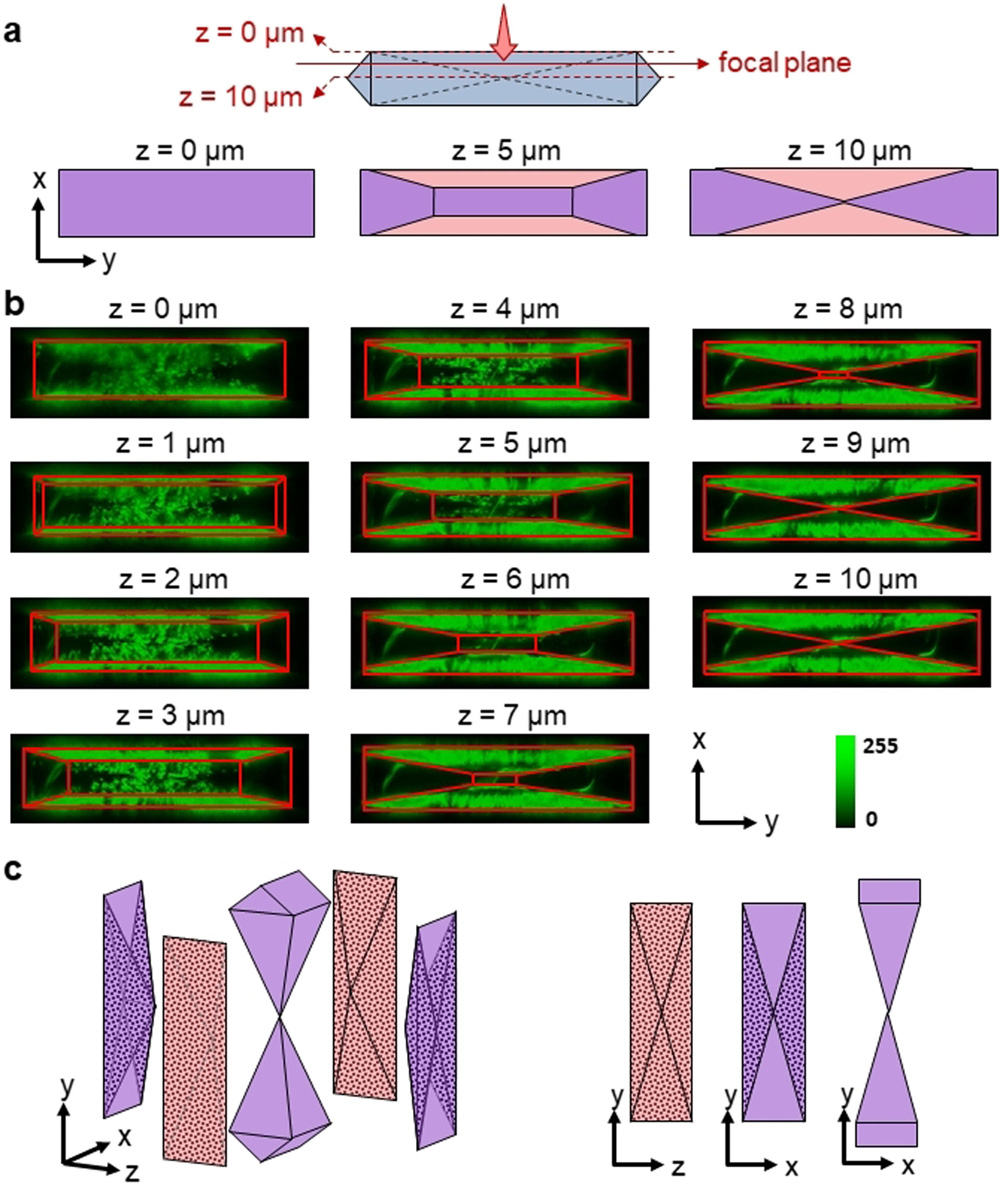
a) confocal *xy*‐raster scanning of ZSM‐5 (roof view) at different *z*‐heights by moving the focal plane from *z*=0*–*10 μm (Δ*z*=1 μm). *xy*‐cuts at *z*=0, 5 and 10 μm show intersection of focal plane with crystal subunits. Color code is identical as in Figure 1; b) *xy*‐maps at *z*=0*–*10 μm; subunit boundaries are indicated as red lines; c) 3D representation of the volume parts of ZSM‐5 which develop hierarchical porosity during desilication (left, dotted areas represent hierarchical pore regions); 2D projections (right).

Fluorescence microscopy visualizes hierarchical porosity via organic DAMPI stains residing in straight micropores of meso‐/macropore walls.^[21]^ Complementary, SAXS microscopy can directly map and speciate the hierarchical pore properties of ZSM‐5 after desilication, allowing important multimodal imaging^[23]^ (Figure 3 a). By xy‐raster scanning the zeolite ZSM‐5 crystal in roof view, a SAXS pattern is obtained for each xy‐pixel (500×500 nm^2^) owing to e^−^‐density differences in empty meso‐/macropore space and the zeolite framework. By integrating the X‐ray scattering pattern over specific segments and applying data analysis (SI‐S7), the extent of (an)isotropic pore formation can be extracted for target pore size regimes. Repeating this protocol for each xy‐pixel yields (an)isotropic pore maps for different meso‐/macropore size ranges (Figure 3 b,c).

**Figure 3:**
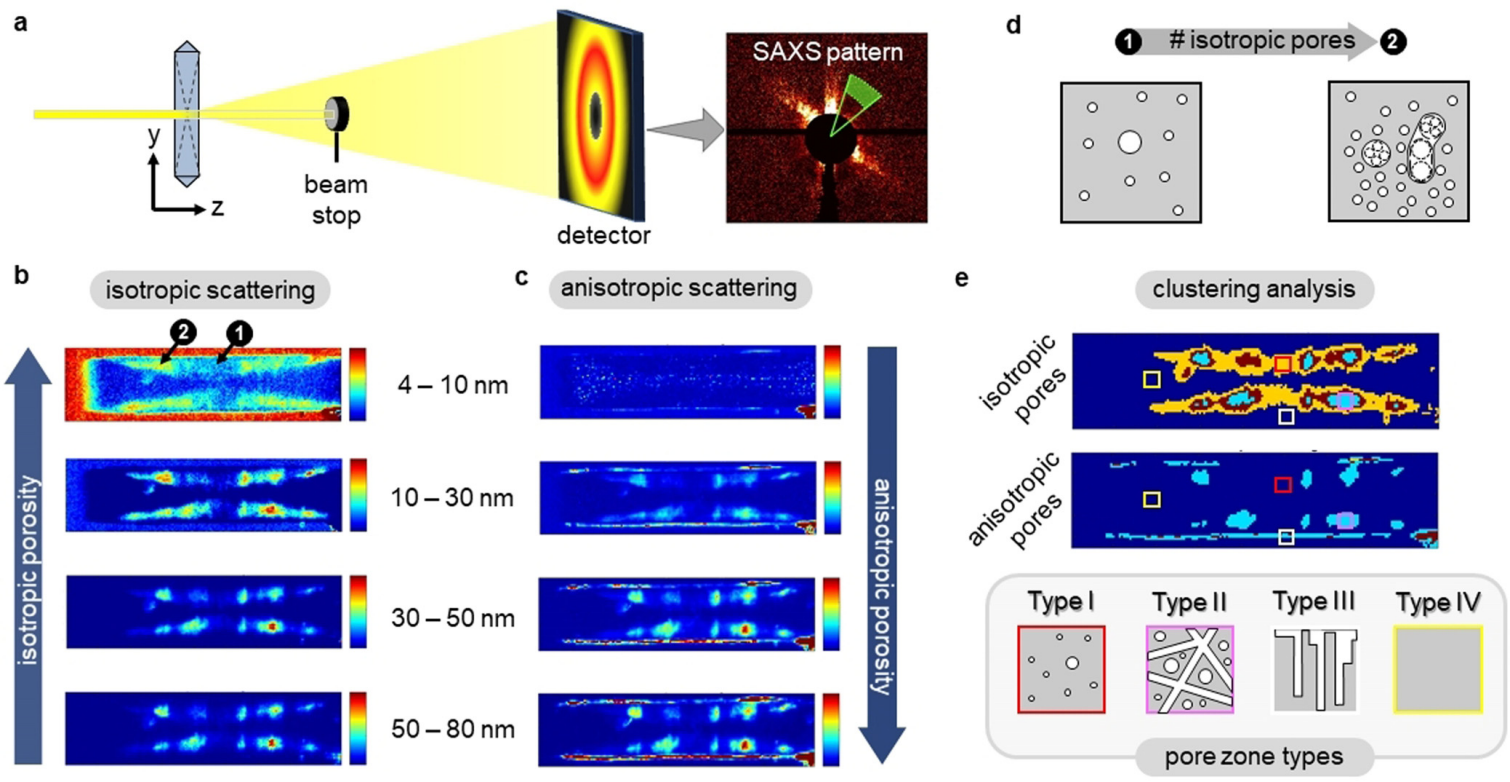
a) SAXS microscopy: 500×500 nm^2^ X‐ray beam xy‐raster scans desilicated ZSM‐5 in roof view. Scattered X‐rays are detected by a 2D‐detector, yielding a SAXS pattern for each xy‐pixel; b) isotropic and c) anisotropic pore distribution maps showing the relative pore abundance within a ZSM‐5 crystal for specific meso‐ (4–10 nm, 10–30 nm, 30–50 nm) and macropore (50–80 nm) size ranges. Two specific locations (1)‐(2) are indicated with low and high pore abundance, respectively, referring to (d); d) representation of region with (1) low and (2) high abundance of small mesopores, in case (2) leading to larger (an)isotropic meso‐ and macropores by pore intergrowth; e) clustering analysis performed on 10–30 nm mesopore maps, and 4 pore zone types.

Opposed to zeolite ZSM‐5 *prior* to desilication (SI‐S8), small isotropic mesopores (4–10 nm) form in triangular projected areas of the crystal only, in accord with 2D projections of fluorescence microscopy (Figure 1 c, Figure 3 b). With increasing pore size, these zones evolve towards more discrete hotspots at locations where small isotropic pores display high abundance. In contrast, anisotropic pores are not present in the small mesopore range and gradually appear as hotspots with increasing pore size (Figure 3 c). These hotspots show strong spatial correlation to hotspots of isotropic macropores. This suggests that large iso‐ and anisotropic pores are generated by pore intergrowth and/or coalescence in regions of high abundance of small isotropic mesopore (Figure 3 d). Furthermore, at the crystal edge, anisotropic porosity is manifested due to defects on the crystal's external surface, enhanced in their intensity by grazing incidence scattering.

The pore characteristics can be classified in four “pore zone types” (Figure 3 e): (Type‐I) triangular projected areas with moderate amounts of isotropic pores, (Type‐II) hotspots in triangular projected areas with high amounts of isotropic as well as disordered anisotropic pores, (Type‐III) the crystal's external surface with high amounts of ordered anisotropic defects, and (Type‐IV) longitudinally shaped zones where no hierarchical porosity develops.

Subunit‐dependent hierarchical porosity formation also appears after steaming‐treatment of ZSM‐5, suggesting a more generally valid phenomenon (SI‐S9). Such intracrystal discrepancies in hierarchical porosity have potentially large implications for diffusion behavior, leading to subunit or even subunit‐segment dependent catalytic performances and deactivation rates.^[4]^ Also, industrial zeolites typically display highly‐intergrown crystallites, implying that the multitude of domains constituting these crystals might exhibit profoundly differing catalytic behaviors.

In conclusion, we have demonstrated the first‐time application of in situ fluorescence microscopy to track the dynamics of hierarchical porosity formation during desilication of zeolite ZSM‐5 on the level of a single‐crystal. In addition, SAXS microscopy is explored as a novel complementary tool to map and speciate the intracrystal meso‐ and macropore properties. This generally‐applicable approach opens perspectives for mapping hierarchical pore properties (in situ) across large crystal volumes in a non‐destructive way, and reveals that intracrystal heterogeneities in hierarchical porosity are more rule than exception, suggesting intracrystal discrepancies in diffusion, catalytic performances and deactivation rates.

## Conflict of interest

The authors declare no conflict of interest.

## Supporting information

As a service to our authors and readers, this journal provides supporting information supplied by the authors. Such materials are peer reviewed and may be re‐organized for online delivery, but are not copy‐edited or typeset. Technical support issues arising from supporting information (other than missing files) should be addressed to the authors.

Supplementary
