# Supplementary material for: Chemical Imaging of Hierarchical Porosity Formation within a Zeolite Crystal Visualized by Small‐Angle X‐Ray Scattering and In‐Situ Fluorescence Microscopy
Source: Angew Chem Int Ed Engl. 2021 May 5;60(25):13803–6. doi: 10.1002/anie.202101747 (PMC8251824; doi:10.1002/anie.202101747)
Supplement: Supplementary file 1 — Supplementary [file ANIE-60-13803-s001.pdf]

Angewandte Chemie
Eine Zeitschrift der Gesellschaft Deutscher Chemiker

Supporting Information

# Chemical Imaging of Hierarchical Porosity Formation within a Zeolite Crystal Visualized by Small-Angle X-Ray Scattering and In-Situ Fluorescence Microscopy

*Matthias Filez, Martin Vesely, Ivan Garcia-Torregrosa, Marianna Gambino, Özgün Attila, Florian Meirer, Eugene A. Katrukha, Maarten B. J. Roeffaers, Jan Garrevoet, Lukas C. Kapitein, and Bert M. Weckhuysen**

anie_202101747_sm_miscellaneous_information.pdf

**Contents**

S1. ZSM-5 zeolite crystals and desilication protocol ........ 2
S2. Confocal fluorescence microscopy ........ 4
S3. Pore front propagation ........ 6
S4. DAMPI diffusion in parent zeolite ZSM-5 crystal ........ 7
S5. Diffusion of DAMPI after hierarchical porosity formation in zeolite ZSM-5 ........ 8
S6. Interpretation of fluorescence maps and 3D reconstructions ........ 9
S7. SAXS microscopy: experiments and data extraction ........ 11
S8. SAXS microscopy of ZSM-5 prior to desilication ........ 15
S9. Hierarchical porosity formation after steaming treatment of zeolite ZSM-5 ........ 16
S10. References ........ 17

## S1. ZSM-5 zeolite crystals and desilication protocol

Large coffin-shaped zeolite ZSM-5 crystals (dimensions = ~ 20×20×100 $\mu m^3$, Si/Al ratio = 17) have been provided by ExxonMobil (Machelen, Belgium). The preparation procedure has been reported elsewhere.[1] The tetrapropylammonium (TPA) template molecules were carefully removed by calcination treatment (heating ramp of 1 K/min) at 823 K for 8 h. After template removal, the zeolite crystals were converted into their acidic form by triple ion-exchange with 10 wt% ammonium nitrate (99+ %, Acros Organics) at 353 K. Thereafter, the zeolites were washed in a 2 M oxalic acid-$H_2O$ (mild) solution and $H_2O$ at room temperature, after which they were subjected to a 6 h calcination treatment at 773 K.

In order to fixate the zeolite crystals on a microscopy coverslip for *in-situ* studies, the recuperated crystal powder was sprinkled on the microscopy coverslip and calcined in the oven for 3 h at 773 K (1 K/min heating rate). After this treatment, the microscopy coverslip was blown with a $N_2$ gun under a fume-hood in order to remove the non-interacting crystals from the substrate. *In-situ* reaction monitoring under the microscope was done by mounting a gene frame cell (25 µL, 1 × 1 $cm^2$, Thermo Scientific) on top of the microscopy coverslip (Figure S1.1). This gene frame cell was positioned around the location where zeolite crystals were visually located after the fixation treatment. The cell is constructed of a gene frame that contains adhesives on both sides, as used for biological applications. One side of the gene frame cell was glued to the microscopy coverslip, whereas the other adhesive side adheres to a glass which closes the reactor after adding the basic solution for desilication.

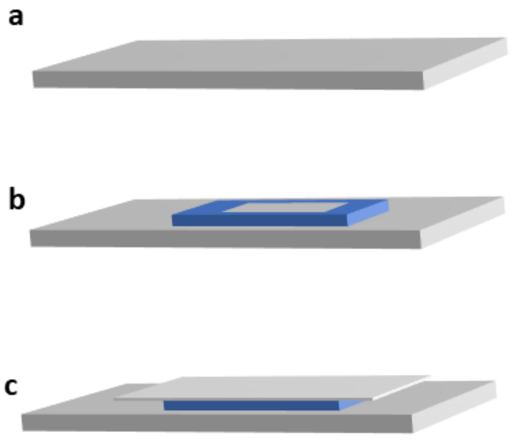


**Figure S1.1.** (a) microscopy coverslip, on which zeolite crystals are fixed on the microscopic scale, (b) gene frame cell which is glued with one side on the microscopy coverslip. From the open top of the gene frame cell, the base solution for desilication is added, which also contains fluorescent DAMPI probes. After adding this solution, (c) a cover glass closes the reactor containing the crystals and base solution using the second adhesive side of the gene frame cell.

In order to perform *in-situ* confocal fluorescence microscopy experiments, 2 mL of an ethanolic 4 µM DAMPI (4-(4-diethylaminostyryl)-N-methylpyridinium iodide) fluorescent solution was added to 2 mL of a 4 M NaOH-$H_2O$ solution. Therefore, after mixing both solutions, a 2 M

NaOH solution with 2 µM DAMPI concentration was obtained. Both solutions were preheated at 40 ºC prior to mixing and subsequent desilication treatment. DAMPI probes are employed to image adsorption of these molecules at the entrance of straight zeolite pore channels.[2] DAMPI has a stilbene-like backbone and is quasi non-fluorescent in solution due to cis-trans isomerizations. The chromophore's emissive properties are recovered when the cis-trans isomerization is sterically prevented, for example, by a sterically narrow fit in a confined space such as a zeolite pore. Thus, DAMPI can selectively visualize those regions of the crystal surface which give access to the straight channels of MFI-type zeolites. As a result of the size of the diethylamino group, further intrusion of this dye into the intact 10-MR framework is very unlikely.

## S2. Confocal fluorescence microscopy

*In-situ* confocal fluorescence microscopy experiments were performed on a Leica TCS SP8 STED3X SMD FLIM around which a Ludin Cube and Box is built for temperature control to 40 °C. A confocal module was available with a filter free spectral detector and HyD detectors. Confocal fluorescence microscopy experiments were performed with a 488 nm laser (circular polarization) focused on the sample with a 100x oil immersed objective and single photon counting detector, before which gating was used in order to reduce the signal-to-noise level of the recorded fluorescence signal. More information on the details of the setup can be found on the following link: https://downloads.leica-microsystems.com/Leica%20TCS%20SP8/Brochures/Leica%20TCS%20SP8%20Scan%20Head-Flyer_EN.pdf.

The in-situ fluorescence microscopy experiments are performed in roof view only, since rotating the single crystal to gable view during the desilication process is not possible, not allowing to collect – and thus here present – the requested complementary data on the gable view. With this experimental limitation in mind, the choice of imaging either 'roof' or 'gable' view during the in-situ fluorescence microscopy experiments has been thought-through prior to the experiment. For the in-situ experiments in roof view, the hierarchical porosity formation in all subunits can be unambiguously assessed, given the considerations below.

1. In gable view, the straight pores containing the fluorescent DAMPI probes in the (100)' pyramidal subunits (Fig. 1,pink) run parallell to the external surface, and perpendicular to the beam directions, while the straight pores in the other four (100)-oriented subunits (Fig. 1,purple) run in the beam direction. Therefore, gable view in principle only allows to image meso- and macropore formation in the (100)' pyramidal subunits. In contrast, roof view allows to image meso- and macropore formation in the four (100) subunits (Fig. 1, purple), since straight pores run perpendicular to the beam direction, but is in principle not sensitive to hierarchical porosity formation in the (100)' pyramidal subunits. However, in the data collected in roof view, we do observe porosity formation in these (100)' pyramidal subunits, which results from strong pore hierarchization, leading to local crystal domain distortions/orientation chances and the corresponding fluorescence response. For these reasons, we do capture information from both (100)'-oriented subunits as well as the four (100) subunits in a single roof shot, thus certifying that roof view is the best choice, and in principle, gable view recording is redundant.
2. Based on swift visual screening in the fluorescence microscope, the (100)' pyramidal subunits exhibit strong fluorescence in gable view, again due to strong meso- and macropore formation in these subunits. Even though the fluorescence microscope

works under confocal conditions, this strong fluorescence in the (100)' subunits generates a strong background when imaging in low-fluorescent z-slices of the crystal. This does not allow to image with high sensitivity in the remaining four (100) subunits.

## S3. Pore front propagation

**Calculation of pore front position in gable subunits relative to crystal surface.**

For calculating the average position of the pore front in the gable pyramidal subunits (see definition in Figure 1.a) as a function of desilication time, the z-slice at z = 10 µm in the middle of the crystal in side view was analyzed for each recorded map (e.g. Figure S3.1 (top)). The gable pyramidal subunits show fluorescence intensity owing to hierarchical porosity formation. Upon time increase, the porosity front moves towards the crystal core. When the pore front reaches the interface region where the pore-rich pyramidal and pore-free longitudonal subunits are intergrown, the pore propagation stops. Therefore, for the calculation of the pore front position, only the regions where the pore front is still propagating to the crystal core and did not reach the intergrowth region is considered. As an example, the red marked areas in Figure S3.1 – up to the red arrows – constitute areas of the crystal where hierarchical porosity formation has halted at the interface between the gable subunits and the longitudinal subunits. Only the non-marked areas, i.e. in between the 4 red arrows, are employed for the calculations. Within this area (see zoomed area in Figure S3.1 (bottom)), 5 random positions are selected where the pore-front-to-surface-distance is calculated, and averaged over the 5 positions. This calculation is done for each data point displayed in Figure 1.d in the main manuscript.

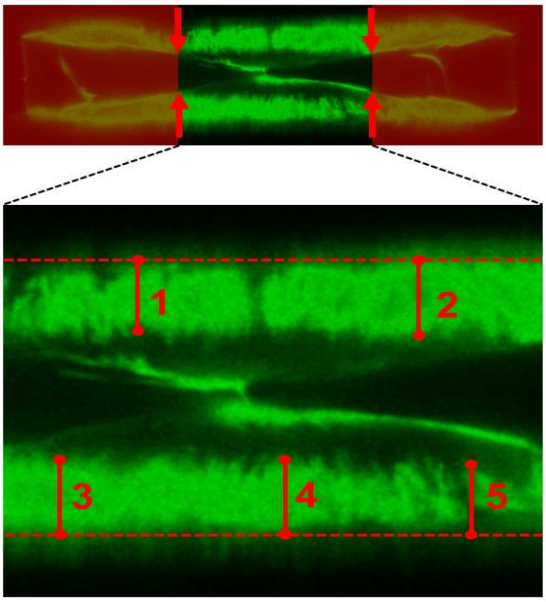


**Figure S3.1.** (top) z-slice at z = 10 µm of the crystal positioned in side view. The red areas are not used for calculation of the pore front position and propagation rate. (bottom) 5 random positions are used for calculating the pore front position in the gable subunits relative to their external surface.

**Propagation rate of the pore front**

The propagation rate is calculated by calculating the numerical derivative of the pore front positions as a function of time. The trend lines displayed in Figure 1d correspond to the best cubic spline fit with pre-selected stiffness to converge to the data.

## S4. DAMPI diffusion in parent zeolite ZSM-5 crystal

Figure S4.1 shows the diffusion dynamics of DAMPI probes into parent ZSM-5. A ZSM-5 crystal was positioned in the beam position, after which a DAMPI solution was added without the addition of NaOH. Measurements after 0, 2 and 12 hours show that the intensity slightly increases in specific defect zones of the crystal where initial fluorescence is present. However, no additional regions of fluorescence are observed, showing that no additional porosity is formed. DAMPI probes thus only get access to porosity accessible via the external surface. It should be noted also that the maximal intensity in Figure S4.1 is 20 counts, while in Fig. 1c amounts to 255. Therefore, the fluorescence intensity in parent ZSM-5 is an order of magnitude smaller compared to the fluorescent intensity observed during hierarchical porosity formation, again supporting no gradual observations of hierarchical porosity formation.

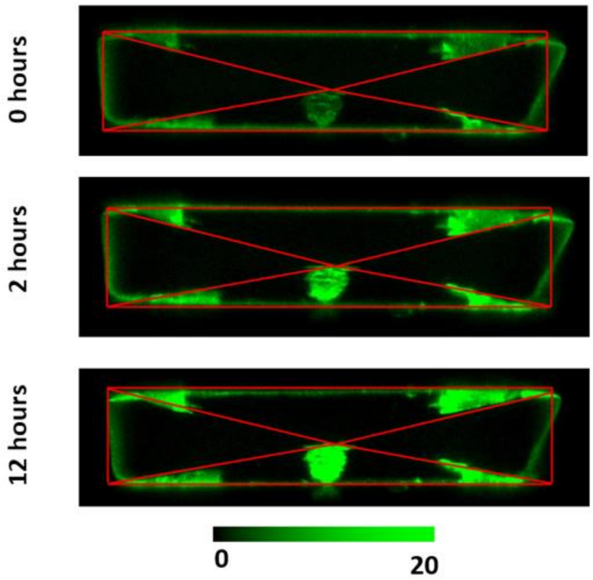


**Figure S4.1.** 2D *in-situ* fluorescence roof projections averaged over all z-heights of the crystal. DAMPI diffusion in a zeolite ZSM-5 crystal without adding NaOH to the watery solution.

## S5. Diffusion of DAMPI after hierarchical porosity formation in zeolite ZSM-5

Figure S5.1 show the diffusion dynamics of DAMPI probes into hierarchically porous zeolite ZSM-5. A ZSM-5 crystal with pre-formed hierarchical porosity was positioned in the beam position, after which a DAMPI solution was added. Measurements after 1 min, 2 min and 102 min show that transient diffusion takes place in the first 2 minutes adter adding the DAMPI probes. However, after 2 minutes, the staining intensity becomes constant, showing that diffusion is complete.

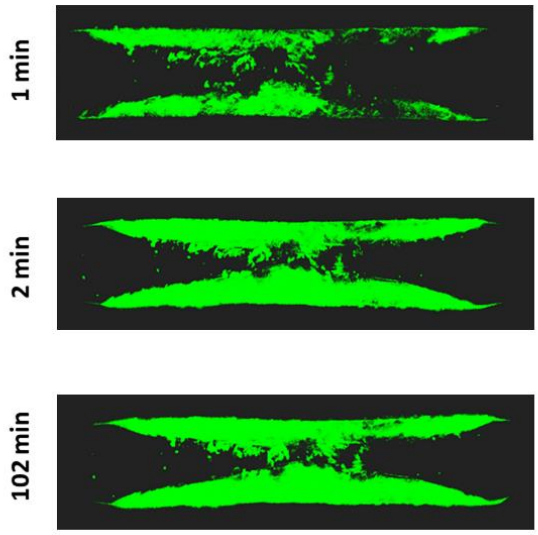


**Figure S5.1.** 2D *in-situ* fluorescence roof projections averaged over all z-heights of the crystal. DAMPI diffusion in a zeolite ZSM-5 crystal which has pre-formed hierarchical porosity.

## S6. Interpretation of fluorescence maps and 3D reconstructions

**Interpretation.** The zeolite ZSM-5 crystals under consideration are constituted of 6 subunits, as illustrated in Figure 1a. DAMPI probes only fit into the straight pores of the micropore network of ZSM-5. However, the DAMPI molecule cannot enter the straight 5.6 Å channels due to its diethylamino end group (7.2 Å), which sterically hinders its inward diffusion. When a zeolite ZSM-5 crystal is positioned in side view under the fluorescence microscope (Figure 1a), the straight channels are perpendicular to the beam direction (i.e. in the x-direction). This allows to excite DAMPI probes in the straight channels of the purple subunits, given the use of a circularly polarized laser. In contrast, the straight channels in the pink subunits in Figure 1a are along the beam direction (i.e. in z-direction), making it impossible to excite DAMPI probes in the micropore's straight channels of the gable (pink) subunits. The strong fluorescense intensity in Figure 2b is therefore caused by (partial) amorphization of the original microporous framework, leading to misoriented straight channels, as a result of extra framework meso- and/or macroporosity formation.

**3D reconstructions.** 3D reconstructions of the crystal volume containing a threshold value of fluorescence intensity – and therefore hierarchical porosity – are shown in Figure S6.1. A cut has been made through the crystal at z = 10 µm to visualize hierarchical porosity from the interior of the half-crystal. When comparing the roof and gable views, it is clearly observed that (i) the pyramidal (100)' subunit in gable view displays hierarchical porosity across its entire volume, (ii) no hierarchical porosity is observed in the longitudonal prism-type volumes in roof and roof-gable view, these prism-type volumes including both longitudinal subunits and segments of the (100)-oriented pyramidal subunits. This implies that (a) the longitudonal subunits are pore-free and (b) only selected segments of the (100) pyramidal subunits are meso- and macropore free, while other are not, further supporting the validity of the representation shown in Fig. 2c. (100)' pyramidal subunits therefore show a different hierarchical porosity pattern compared to (100) pyramidal subunits.

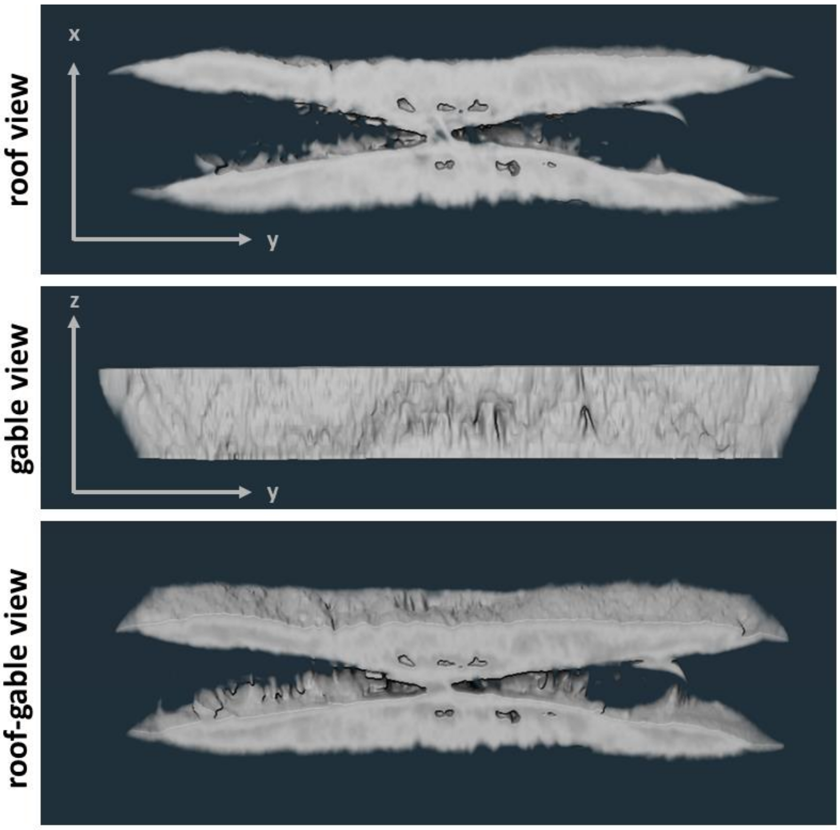


**Figure S6.1.** 3D reconstruction of hierarchically porous volume in a ZSM-5 crystal at a late stage of in-situ desilication. Roof, gable and a rotated roof-gable view are shown.

## S7. SAXS microscopy: experiments and data extraction

### Experiments

SAXS microscopy data were collected using a Eiger X 4M hybrid pixel detector (Dectris Ltd.) at the Hard X-ray Micro/Nano-Probe beamline P06 at the PETRA III synchrotron facility, which is part of DESY. A single zeolite ZSM-5 crystal has been attached on a needle, which is glued in a goniometer pin at home. This goniometer pin was then inserted in the goniometer head at P06. This allowed proper alignement of the single crystal in the beam position and center of rotation, required rotation into side view. Once positioned in side view, transmission SAXS microscopy maps were recorded with 500 x 500 $nm^2$ pixel beam size at 12 keV with a sample-to-detector distance of 2 m.

### Data extraction

SAXS microscopy data extracting is done by using in-house generated MATLAB scripts. The overall principle behind the data analysis procedure is based on scattering contrast within specific segments of the SAXS pattern. By applying a SAXS analysis workflow showing similarities to the validated methodologies of Bunk et al.[3], Liebi et al.[4] and Schaff et al.[5], azimutal plots or segmental profiles can be generated which contain pore information (*vide infra*). Thus, the data extraction does not rely on conventional Porod/Guinier SAXS analysis used regularly for data analysis.

**Pre-processing.**

Several steps were involved in pre-processing of SAXS patterns. First, a mask was generated. This mask included the locations where (1) no detector pixels were present, (2) death pixels were manifested (e.g. high intensity outliers) and (3) where the beamstop was located. The mask values were set to NaN, while non-masked pixels were set to 1. By multiplying the mask to the original SAXS pattern, all non-valid detector pixels were set to NaN, which allowed easy data processing, such as integration over all non-NaN valued pixels. The SAXS pattern was then centered around the beam position and calibrated for its detector-to-sample position.

**Azimutal plots and probability distributions**

For each xy-pixel in the SAXS microscopy measurement (Figure 3a), a SAXS pattern is collected (Figure S6.1a), containing all hierarchical pore information to be extracted. By azimuthal ($\theta$) and radial ($r$) integration in a specific range ($\iint dr \cdot d\theta$), e.g. within the green area in Figure S7.1a, and repeating this integration for different azimuthal segments over the full $\theta$ = $0 \rightarrow 2\pi$ range, an 'azimuthal profile' can be obtained for a pre-selected radial range $[r_1, r_2]$. The total number of azimutal segments amounts to 512, in order to obtain high angular resolution in the azimutal plot, while the radial ranges were subdivided in the four regions of

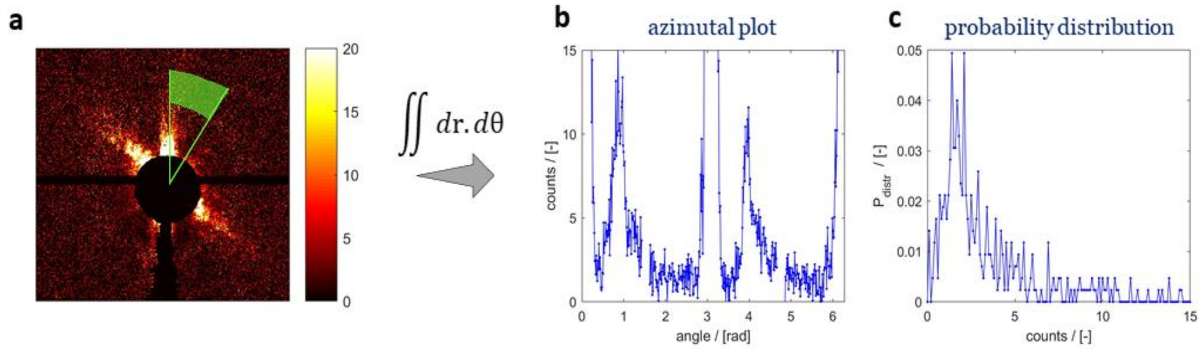


**Figure S7.1.** (a) SAXS pattern. (b) segments of the SAXS pattern are azimutally and radially integrated (within the green marked area in (a)) to obtain azimutal plots for specific radial segments, as well as a (c) probability distribution of counts. See the text for more details.

interest: 4-10 nm, 10-30 nm, 30-50 nm and 50-80 nm. This azimutal plot contains the number of detected X-rays per angular segment as a function of the azimuthal scattering angle within a pore size range $[r_1, r_2]$ (Figure S7.1b). Physically, the azimutal plot displays in which angular regions X-ray scattering is observed in the SAXS pattern for specific radial ranges. For example, the SAXS pattern in Figure S7.1a shows two symmetric ‘flares’ around the beamstop. These flares result into four intense peaks (2 pairs which are $\pi$ radials apart) in the azimutal plot after segmentation/integration in specific radial ranges. As a next step, a ‘probability distribution’ plot can be extracted from this azimutal plot which displays the probality of observing a segment with a certain number of X-ray counts (*vide infra*).

These two plots (Figure S7.1b,c) can yield more insight into the meso- and macropore properties of each xy-pixel recorded during SAXS microscopy, following from the coming considerations. Hierarchical pores have either isotropic (e.g. spherical) or anisotropic (e.g. ellipsoidal) pores. Isotropic pores lead to isotropic scattering in the SAXS pattern, implying a similar number of X-ray counts over all azimuthal angles. In contrast, anisotropic pores scatter anisotropically, generating anisotropic scattering ‘flares’ in specific angular ranges of the SAXS pattern (e.g. the flares in Figure S7.1a). As a consequence, isotropic scattering leads to an increase in the background of the azimutal profile, specifically a height increase in the minimum of the azimuthal profile, while anisotropic scattering generates sharp peak features in the azimutal profile. These sharp features shape the probability distribution via its (1) broadness, forming a measure for the extent of anisotropic pore formation, and (2) skewness, forming a measure for the degree of order of anisotropic pores.

To illustrate the above statements, Figure S7.2 shows three showcases which exhibit significantly different azimutal plots, probability distributions and thus different pore structure. In Case I, (1) the azimutal profile shows a low minimum, i.e. low number of isotropic pores, (2) small variance, i.e. low number of anisotropic pores, and (3) small skewness of the probability

distribution, i.e. small order of anisotropic pores. Thus, no pores are formed. In Case II, (1) the azimutal profile shows a high minimum, i.e. high number of isotropic pores, (2) large variance, i.e. large number of anisotropic pores, and (3) small skewness of the probability distribution, i.e. small order of anisotropic pores. Therefore, both isotropic and anisotropic pores are formed, the latter begin disordered in their orientation. In Case III, (1) the azimutal profile shows a small minimum, i.e. small number of isotropic pores, (2) large variance, i.e. large number of anisotropic pores, and (3) high skewness of the probability distribution, i.e. high order of anisotropic pores. Therefore, only anisotropic pores are formed, being highly ordered in their orientation.

An important point of caution is associated with the analysis methods and its interpretation. Particularly, flares generated by scattering of many randomly oriented anisotropic pores result in averaging and leads to an isotropic scattering signal. In the observed data, however, a sharp contrast is observed between (i) SAXS patterns which contain discontinuous – and often highly intense – flares and associated discontinuous/intense peak features in the azimutal profile on the one hand, and (ii) continuous isotropic SAXS patterns which display flare-free images and show highly isotropic signals on the other hand. This observation strengthens the workflow of the analysis methodology and interpretation, since discontinuous anisotropic contributions to the signal have clearly different signal 'fingerprints' compared to the continuous and isotropic background – already observable by visual analysis. It is thus necessary and important to keep in mind this assumption/simplification upon interpretation of the analysis output.

In the specific Case II of Figure S7.2, the azimutal profile shows clear signs of multimodality in the azimutal profile, pointing towards anisotropic pores which are oriented in specific directions. However, these directions are rather random when comparing different subsequent pixels, learning that indeed anisotropic pores are present, but in a rather disordered fashion/random orientation. This finding highlights the true power of applying microscopy: on the bulk scale, such observations might not be possible, since over large volumes, the signals would be statistically averaged to form a fully 'horizontal' isotropic signal.

**(An)isotropic pore distribution maps and clustering analysis**

By automated repetition of the above-explained analysis procedure – i.e. the calculation of azimuthal plots and probability distributions of a single SAXS pattern – for each xy-pixel, isotropic and anisotropic pore distribution maps can be obtained, as displayed in Figure 3.b for different meso- and macropore size ranges.

In order to classify these pore distribution maps in different areas where similar pore properties are manifested, clustering analysis was done via MATLAB scripting. Therefore, four 'clusters' were used for partitioning the sample data into groups, resulting into a sufficient number of

classes for achieving rigid segmentation analysis. From this clustering analysis, four pore zone types are also recognized, as explained in the main text and illustrated in Figure 3e.

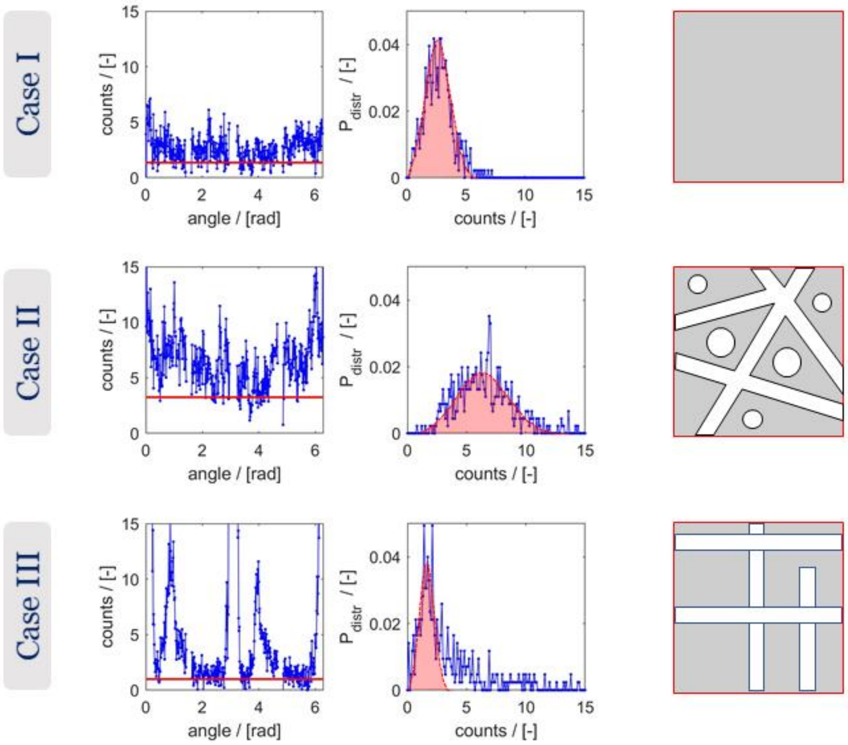


**Figure S7.2.** Developed SAXS data analysis. Three showcases showing (left) azimuthal plots, (middle) probability distributions and (right) a schematic representation of the corresponding hierarchical pore structure. The three cases presented are resulting from real SAXS data analysis. The red-shaded areas (middle) in the probability distributions are (symmetric) gaussians, which are fit to the low range = [0,max($P_{distr}$)] of the probability distribution. This gaussian is solely a guide for the eye not involved in quantification of the data, but can visualize the skewness of the probability distribution, which is a measure of the order of the anisotropic pores. For example, in Case III, non-zero probabilities are clearly observed for higher count numbers, showcasing high order of the anisotropic pores, as schematically detailed in the figure on the bottom right.

## S8. SAXS microscopy of ZSM-5 prior to desilication

Figure S8.1 displays the pore properties of a zeolite ZSM-5 crystal prior to desilication, showing no significant internal hierarchical porosity. The edges of the crystal do show some X-ray scattering intensity as a consequence of the inherent defective nature of the surface of a ZSM-5 crystal.

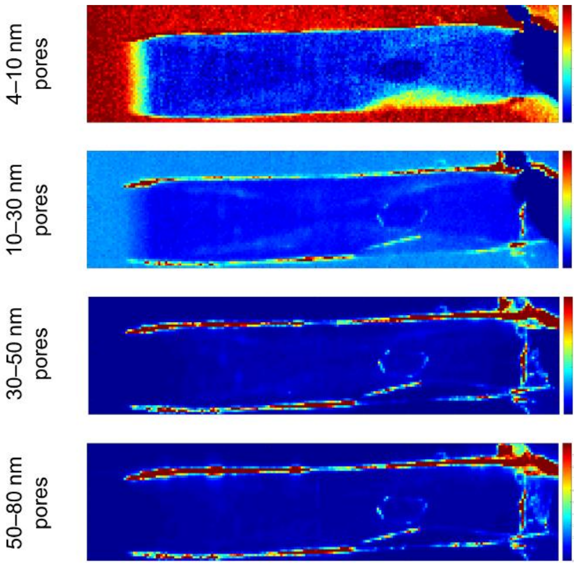


**Figure S8.1.** SAXS microscopy maps of zeolite ZSM-5 crystal prior to desilication, including both isotropic as well as anisotropic contributions. The pore ranges for each image are indicated.

## S9. Hierarchical porosity formation after steaming treatment of zeolite ZSM-5

In order to perform steaming treatment, zeolites were placed in a tubular flow oven and heated to 700°C with a heating rate of 5 °C/min. For the steaming treatment, a water-saturated (373°C) $N_2$ flow (150 mL/min) was used for 5 h. Figure S9.1 shows the fluorescence xy-maps from z = 0-8 µm for a steamed zeolite ZSM-5 cyrstal in side view (as in Figure 2b) in which DAMPI probes were used for staining. The regions in which hierarchical porosity has developed as a result of steaming are very similar to desilication. Particularly, the xy-map at z = 0 µm show mirrored triangular regions in which hierarchical porosity is observed, similar to the z = 0 µm plane of desilicated crystals in Figure 2b.

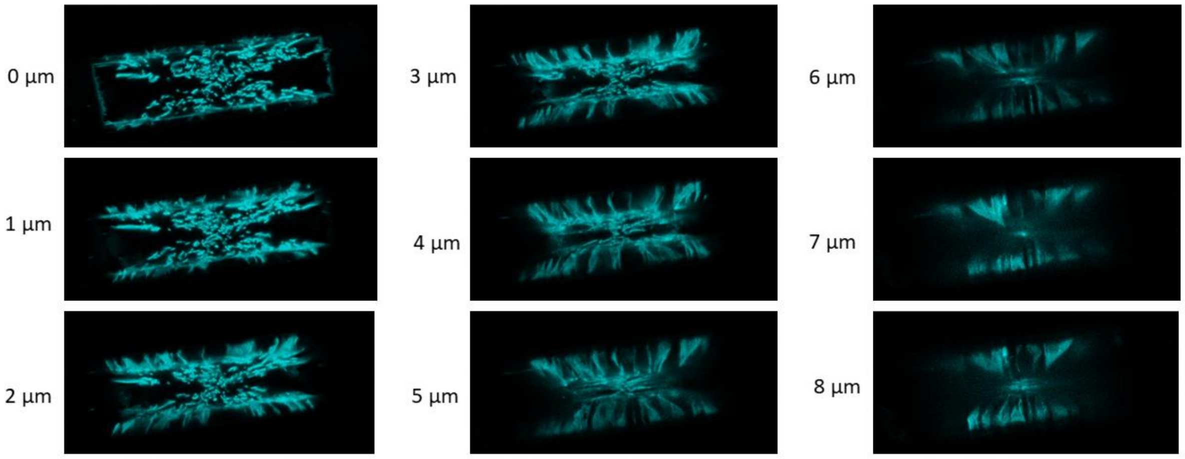


**Figure S9.1.** Fluorescence microscopy xy-maps at z-heights from 0-8 µm by DAMPI staining after steaming treatment of zeolite ZSM-5.

## S10. References


[1] L. R. Aramburo, L. Karwacki, P. Cubillas, S. Asahina, D. A. M. de Winter, M. R. Drury, I. L. C. Buurmans, E. Stavitski, D. Mores, M. Daturi, P. Bazin, P.; Dumas, F. Thibault-Starzyk, J. A. Post, M. W. Anderson, O. Terasaki, B. M. Weckhuysen, *Chem. Eur. J.* **2011**, *17*, 13773–13781.

[2] M. B. J. Roeffaers, R. Ameloot, M. Baruah, H. Uji-i, M. Bulut, G. De Cremer, U. Müller, P. A. Jacobs, J. Hofkens, B. F. Sels, D. E. De Vos, *J. Am. Chem. Soc.* **2008**, *130*, 5763–5772.

[3] O. Bunk, M. Bech, T. H. Jensen, R. Feidenhans'l, T. Binderup, A. Menzel, F. Pfeiffer, *New J. Phys.* **2009**, *11*, 123016.

[4] M. Liebi, M. Georgiadis, A. Menzel, P. Schneider, J. Kohlbrecher, O. Bunk, M. Guizar-Sicairos, *Nature* **2015**, *527*, 349.

[5] F. Schaff, M. Bech, P. Zaslansky, C. Jud, M. Liebi, M. Guizar-Sicairos, F. Pfeiffer, *Nature* **2015**, *527*, 353.
